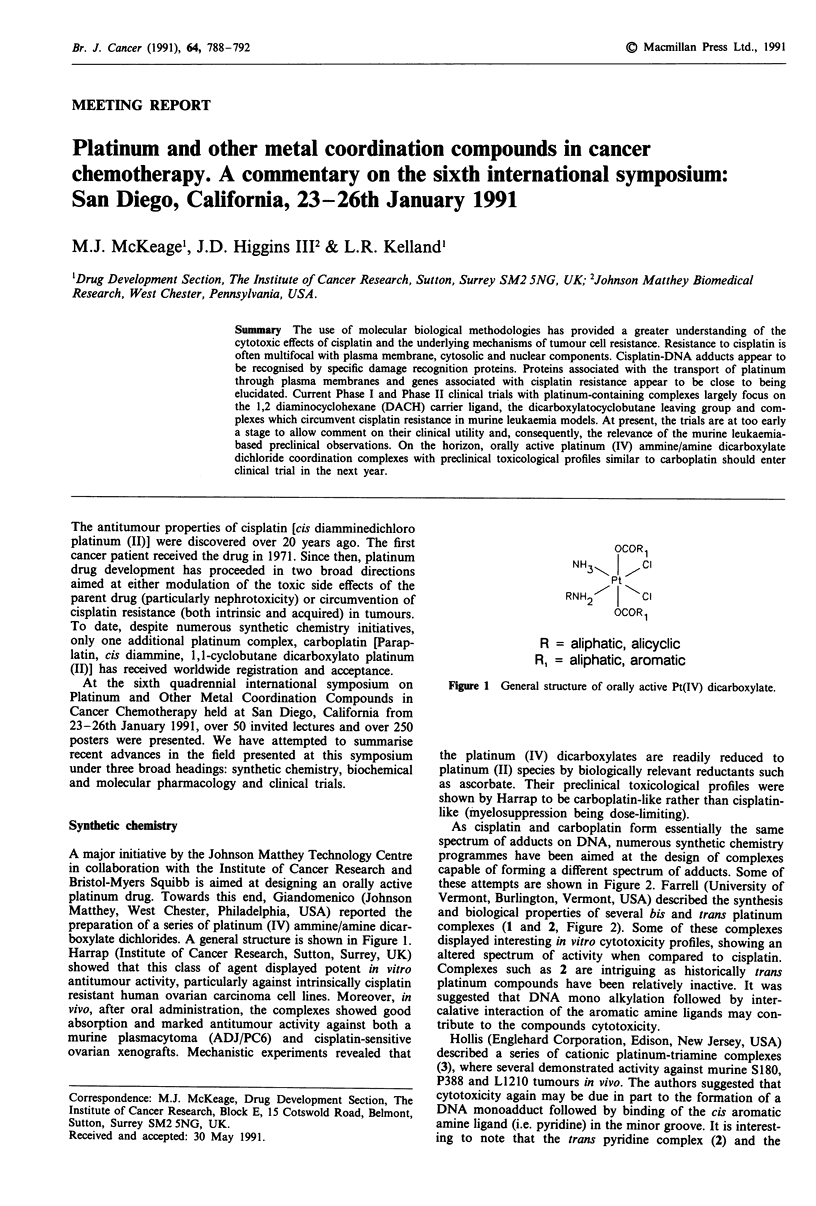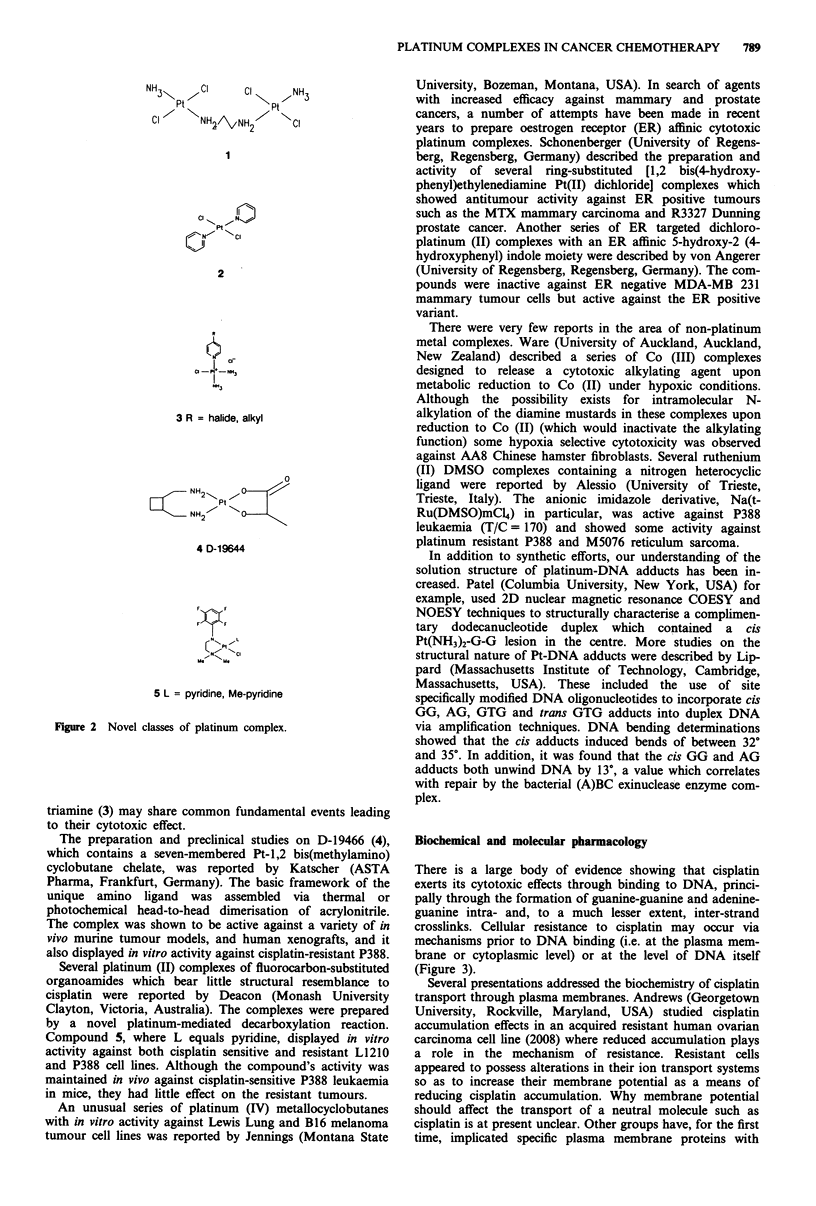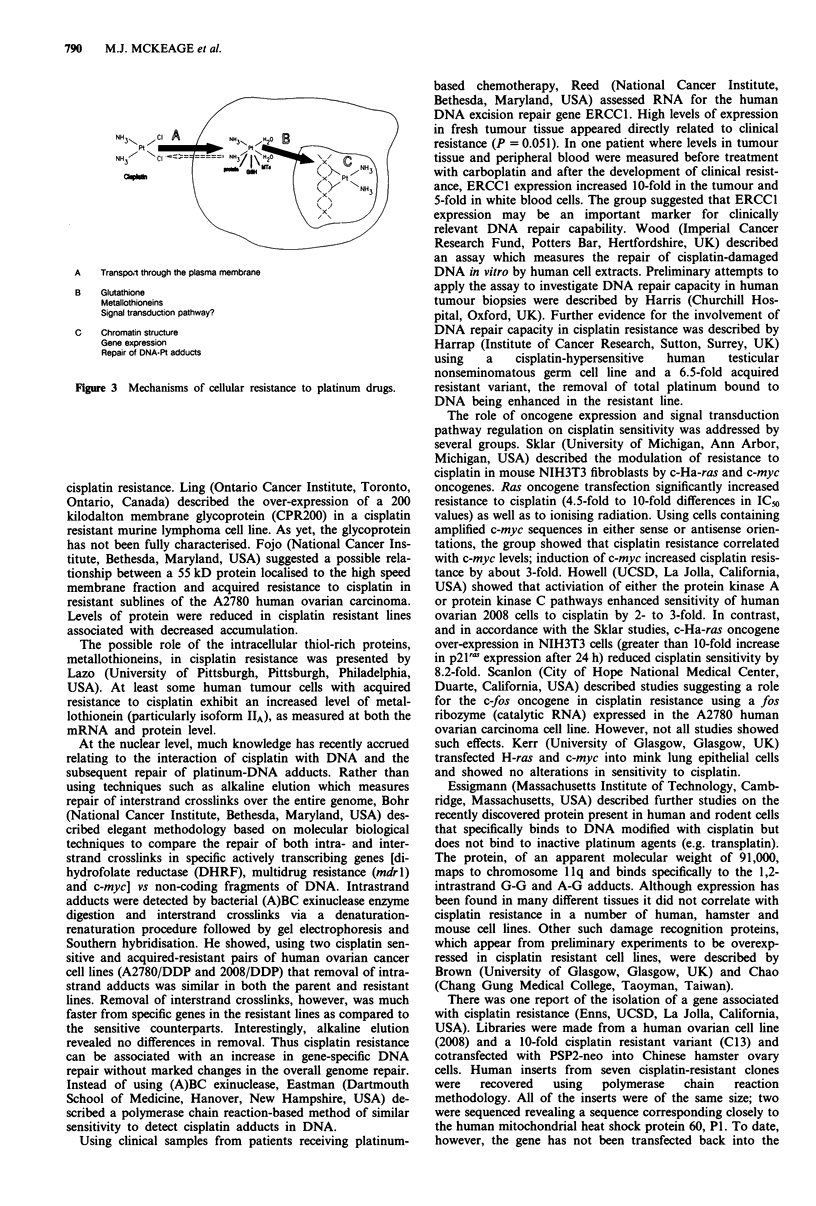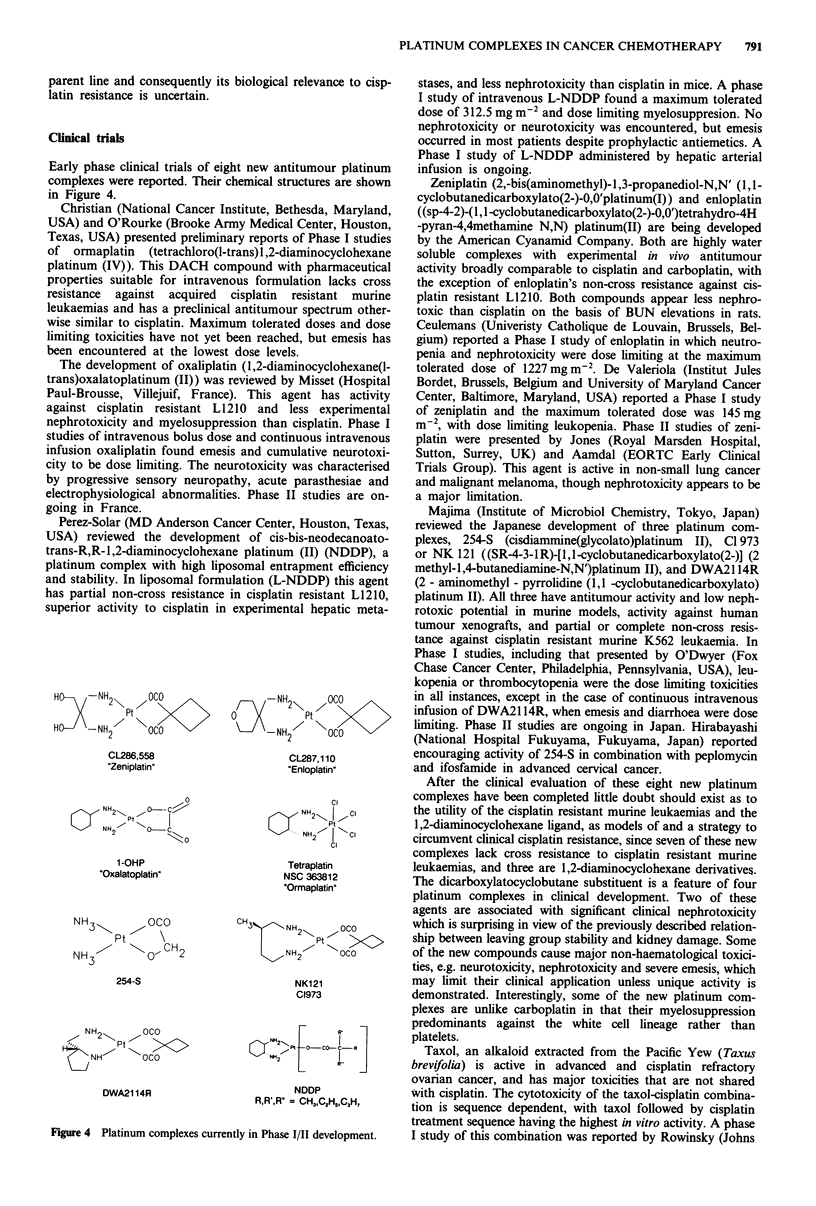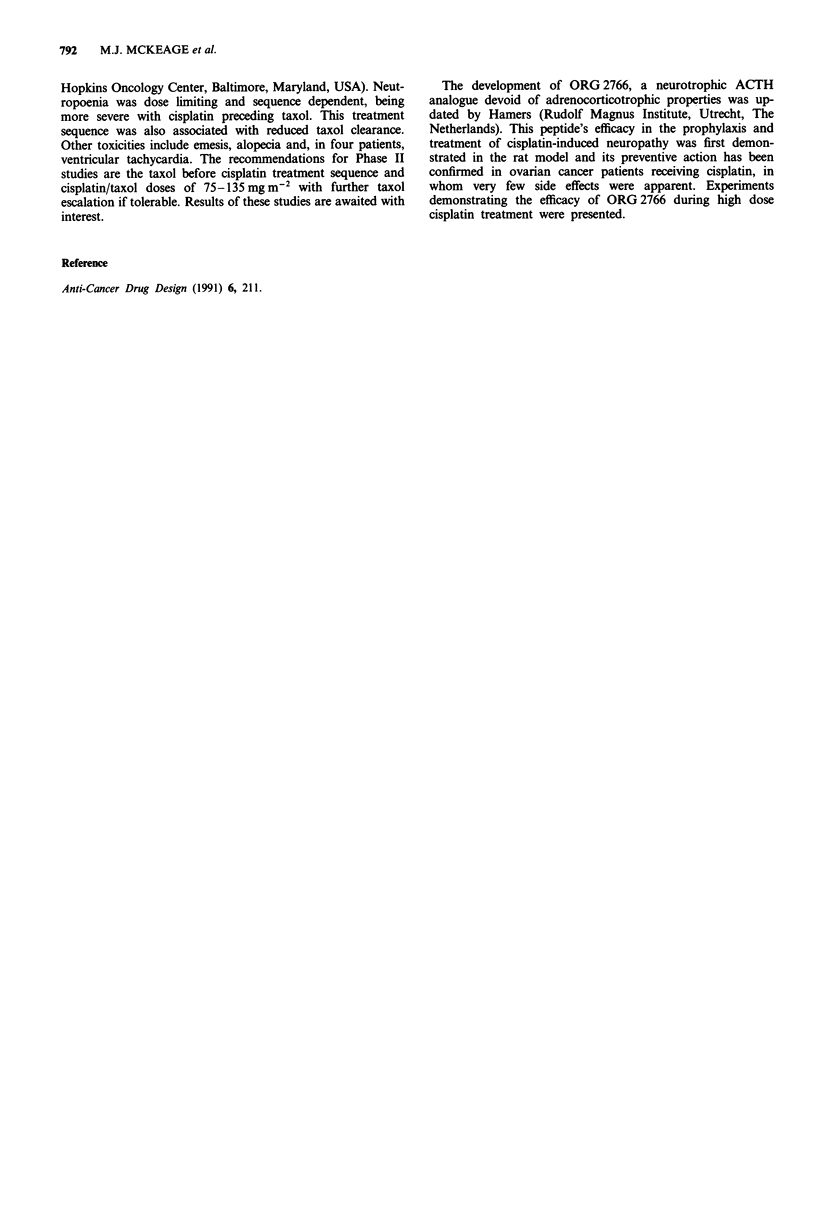# Platinum and other metal coordination compounds in cancer chemotherapy. A commentary on the sixth international symposium: San Diego, California, 23-26th January 1991.

**DOI:** 10.1038/bjc.1991.400

**Published:** 1991-10

**Authors:** M. J. McKeage, J. D. Higgins, L. R. Kelland

**Affiliations:** Drug Development Section, Institute of Cancer Research, Sutton, Surrey, UK.

## Abstract

The use of molecular biological methodologies has provided a greater understanding of the cytotoxic effects of cisplatin and the underlying mechanisms of tumour cell resistance. Resistance to cisplatin is often multifocal with plasma membrane, cytosolic and nuclear components. Cisplatin-DNA adducts appear to be recognised by specific damage recognition proteins. Proteins associated with the transport of platinum through plasma membranes and genes associated with cisplatin resistance appear to be close to being elucidated. Current Phase I and Phase II clinical trials with platinum-containing complexes largely focus on the 1,2 diaminocyclohexane (DACH) carrier ligand, the dicarboxylatocyclobutane leaving group and complexes which circumvent cisplatin resistance in murine leukaemia models. At present, the trials are at too early a stage to allow comment on their clinical utility and, consequently, the relevance of the murine leukaemia-based preclinical observations. On the horizon, orally active platinum (IV) ammine/amine dicarboxylate dichloride coordination complexes with preclinical toxicological profiles similar to carboplatin should enter clinical trial in the next year.


Br. J. Cancer (1991), 64, 788-792 © Macmillan Press Ltd., 1991

MEETING REPORT

### Platinum and other metal coordination compounds in cancer chemotherapy. A commentary on the sixth international symposium: San Diego, California, 23-26th January 1991

M.J. McKeage[1], J.D. Higgins III[2] & L.R. Kelland[1]

[1]*Drug Development Section, The Institute of Cancer Research, Sutton, Surrey SM2 5NG, UK;* [2]*Johnson Matthey Biomedical Research, West Chester, Pennsylvania, USA.*

**Summary** The use of molecular biological methodologies has provided a greater understanding of the cytotoxic effects of cisplatin and the underlying mechanisms of tumour cell resistance. Resistance to cisplatin is often multifocal with plasma membrane, cytosolic and nuclear components. Cisplatin-DNA adducts appear to be recognised by specific damage recognition proteins. Proteins associated with the transport of platinum through plasma membranes and genes associated with cisplatin resistance appear to be close to being elucidated. Current Phase I and Phase II clinical trials with platinum-containing complexes largely focus on the 1,2 diaminocyclohexane (DACH) carrier ligand, the dicarboxylatocyclobutane leaving group and complexes which circumvent cisplatin resistance in murine leukaemia models. At present, the trials are at too early a stage to allow comment on their clinical utility and, consequently, the relevance of the murine leukaemia-based preclinical observations. On the horizon, orally active platinum (IV) ammine/amine dicarboxylate dichloride coordination complexes with preclinical toxicological profiles similar to carboplatin should enter clinical trial in the next year.

The antitumour properties of cisplatin [*cis* diamminedichloro platinum (II)] were discovered over 20 years ago. The first cancer patient received the drug in 1971. Since then, platinum drug development has proceeded in two broad directions aimed at either modulation of the toxic side effects of the parent drug (particularly nephrotoxicity) or circumvention of cisplatin resistance (both intrinsic and acquired) in tumours. To date, despite numerous synthetic chemistry initiatives, only one additional platinum complex, carboplatin [Paraplatin, *cis* diammine, 1,1-cyclobutane dicarboxylato platinum (II)] has received worldwide registration and acceptance.

At the sixth quadrennial international symposium on Platinum and Other Metal Coordination Compounds in Cancer Chemotherapy held at San Diego, California from 23-26th January 1991, over 50 invited lectures and over 250 posters were presented. We have attempted to summarise recent advances in the field presented at this symposium under three broad headings: synthetic chemistry, biochemical and molecular pharmacology and clinical trials.

### Synthetic chemistry

A major initiative by the Johnson Matthey Technology Centre in collaboration with the Institute of Cancer Research and Bristol-Myers Squibb is aimed at designing an orally active platinum drug. Towards this end, Giandomenico (Johnson Matthey, West Chester, Philadelphia, USA) reported the preparation of a series of platinum (IV) ammine/amine dicarboxylate dichlorides. A general structure is shown in Figure 1. Harrap (Institute of Cancer Research, Sutton, Surrey, UK) showed that this class of agent displayed potent *in vitro* antitumour activity, particularly against intrinsically cisplatin resistant human ovarian carcinoma cell lines. Moreover, *in vivo*, after oral administration, the complexes showed good absorption and marked antitumour activity against both a murine plasmacytoma (ADJ/PC6) and cisplatin-sensitive ovarian xenografts. Mechanistic experiments revealed that

R = aliphatic, alicyclic

$R_1$ = aliphatic, aromatic

**Figure 1** General structure of orally active Pt(IV) dicarboxylate.

the platinum (IV) dicarboxylates are readily reduced to platinum (II) species by biologically relevant reductants such as ascorbate. Their preclinical toxicological profiles were shown by Harrap to be carboplatin-like rather than cisplatin-like (myelosuppression being dose-limiting).

As cisplatin and carboplatin form essentially the same spectrum of adducts on DNA, numerous synthetic chemistry programmes have been aimed at the design of complexes capable of forming a different spectrum of adducts. Some of these attempts are shown in Figure 2. Farrell (University of Vermont, Burlington, Vermont, USA) described the synthesis and biological properties of several *bis* and *trans* platinum complexes (**1** and **2**, Figure 2). Some of these complexes displayed interesting *in vitro* cytotoxicity profiles, showing an altered spectrum of activity when compared to cisplatin. Complexes such as **2** are intriguing as historically *trans* platinum compounds have been relatively inactive. It was suggested that DNA mono alkylation followed by intercalative interaction of the aromatic amine ligands may contribute to the compounds cytotoxicity.

Hollis (Englehard Corporation, Edison, New Jersey, USA) described a series of cationic platinum-triamine complexes (**3**), where several demonstrated activity against murine S180, P388 and L1210 tumours *in vivo*. The authors suggested that cytotoxicity again may be due in part to the formation of a DNA monoadduct followed by binding of the *cis* aromatic amine ligand (i.e. pyridine) in the minor groove. It is interesting to note that the *trans* pyridine complex (**2**) and the

Correspondence: M.J. McKeage, Drug Development Section, The Institute of Cancer Research, Block E, 15 Cotswold Road, Belmont, Sutton, Surrey SM2 5NG, UK.
Received and accepted: 30 May 1991.

1

2

3 R = halide, alkyl

4 D-19644

5 L = pyridine, Me-pyridine

**Figure 2** Novel classes of platinum complex.

triamine (3) may share common fundamental events leading to their cytotoxic effect.

The preparation and preclinical studies on D-19466 (4), which contains a seven-membered Pt-1,2 bis(methylamino) cyclobutane chelate, was reported by Katscher (ASTA Pharma, Frankfurt, Germany). The basic framework of the unique amino ligand was assembled via thermal or photochemical head-to-head dimerisation of acrylonitrile. The complex was shown to be active against a variety of *in vivo* murine tumour models, and human xenografts, and it also displayed *in vitro* activity against cisplatin-resistant P388.

Several platinum (II) complexes of fluorocarbon-substituted organoamides which bear little structural resemblance to cisplatin were reported by Deacon (Monash University Clayton, Victoria, Australia). The complexes were prepared by a novel platinum-mediated decarboxylation reaction. Compound 5, where L equals pyridine, displayed *in vitro* activity against both cisplatin sensitive and resistant L1210 and P388 cell lines. Although the compound's activity was maintained *in vivo* against cisplatin-sensitive P388 leukaemia in mice, they had little effect on the resistant tumours.

An unusual series of platinum (IV) metallocyclobutanes with *in vitro* activity against Lewis Lung and B16 melanoma tumour cell lines was reported by Jennings (Montana State University, Bozeman, Montana, USA). In search of agents with increased efficacy against mammary and prostate cancers, a number of attempts have been made in recent years to prepare oestrogen receptor (ER) affinic cytotoxic platinum complexes. Schonenberger (University of Regensberg, Regensberg, Germany) described the preparation and activity of several ring-substituted [1,2 bis(4-hydroxyphenyl)ethylenediamine Pt(II) dichloride] complexes which showed antitumour activity against ER positive tumours such as the MTX mammary carcinoma and R3327 Dunning prostate cancer. Another series of ER targeted dichloroplatinum (II) complexes with an ER affinic 5-hydroxy-2 (4-hydroxyphenyl) indole moiety were described by von Angerer (University of Regensberg, Regensberg, Germany). The compounds were inactive against ER negative MDA-MB 231 mammary tumour cells but active against the ER positive variant.

There were very few reports in the area of non-platinum metal complexes. Ware (University of Auckland, Auckland, New Zealand) described a series of Co (III) complexes designed to release a cytotoxic alkylating agent upon metabolic reduction to Co (II) under hypoxic conditions. Although the possibility exists for intramolecular N-alkylation of the diamine mustards in these complexes upon reduction to Co (II) (which would inactivate the alkylating function) some hypoxia selective cytotoxicity was observed against AA8 Chinese hamster fibroblasts. Several ruthenium (II) DMSO complexes containing a nitrogen heterocyclic ligand were reported by Alessio (University of Trieste, Trieste, Italy). The anionic imidazole derivative, Na(t-Ru(DMSO)$mCl_4$) in particular, was active against P388 leukaemia (T/C = 170) and showed some activity against platinum resistant P388 and M5076 reticulum sarcoma.

In addition to synthetic efforts, our understanding of the solution structure of platinum-DNA adducts has been increased. Patel (Columbia University, New York, USA) for example, used 2D nuclear magnetic resonance COESY and NOESY techniques to structurally characterise a complimentary dodecanucleotide duplex which contained a *cis* $Pt(NH_3)_2$-G-G lesion in the centre. More studies on the structural nature of Pt-DNA adducts were described by Lippard (Massachusetts Institute of Technology, Cambridge, Massachusetts, USA). These included the use of site specifically modified DNA oligonucleotides to incorporate *cis* GG, AG, GTG and *trans* GTG adducts into duplex DNA via amplification techniques. DNA bending determinations showed that the *cis* adducts induced bends of between 32° and 35°. In addition, it was found that the *cis* GG and AG adducts both unwind DNA by 13°, a value which correlates with repair by the bacterial (A)BC exinuclease enzyme complex.

### Biochemical and molecular pharmacology

There is a large body of evidence showing that cisplatin exerts its cytotoxic effects through binding to DNA, principally through the formation of guanine-guanine and adenine-guanine intra- and, to a much lesser extent, inter-strand crosslinks. Cellular resistance to cisplatin may occur via mechanisms prior to DNA binding (i.e. at the plasma membrane or cytoplasmic level) or at the level of DNA itself (Figure 3).

Several presentations addressed the biochemistry of cisplatin transport through plasma membranes. Andrews (Georgetown University, Rockville, Maryland, USA) studied cisplatin accumulation effects in an acquired resistant human ovarian carcinoma cell line (2008) where reduced accumulation plays a role in the mechanism of resistance. Resistant cells appeared to possess alterations in their ion transport systems so as to increase their membrane potential as a means of reducing cisplatin accumulation. Why membrane potential should affect the transport of a neutral molecule such as cisplatin is at present unclear. Other groups have, for the first time, implicated specific plasma membrane proteins with

A Transport through the plasma membrane

B Glutathione
Metallothioneins
Signal transduction pathway?

C Chromatin structure
Gene expression
Repair of DNA-Pt adducts

**Figure 3** Mechanisms of cellular resistance to platinum drugs.

cisplatin resistance. Ling (Ontario Cancer Institute, Toronto, Ontario, Canada) described the over-expression of a 200 kilodalton membrane glycoprotein (CPR200) in a cisplatin resistant murine lymphoma cell line. As yet, the glycoprotein has not been fully characterised. Fojo (National Cancer Institute, Bethesda, Maryland, USA) suggested a possible relationship between a 55 kD protein localised to the high speed membrane fraction and acquired resistance to cisplatin in resistant sublines of the A2780 human ovarian carcinoma. Levels of protein were reduced in cisplatin resistant lines associated with decreased accumulation.

The possible role of the intracellular thiol-rich proteins, metallothioneins, in cisplatin resistance was presented by Lazo (University of Pittsburgh, Pittsburgh, Philadelphia, USA). At least some human tumour cells with acquired resistance to cisplatin exhibit an increased level of metallothionein (particularly isoform $II_A$), as measured at both the mRNA and protein level.

At the nuclear level, much knowledge has recently accrued relating to the interaction of cisplatin with DNA and the subsequent repair of platinum-DNA adducts. Rather than using techniques such as alkaline elution which measures repair of interstrand crosslinks over the entire genome, Bohr (National Cancer Institute, Bethesda, Maryland, USA) described elegant methodology based on molecular biological techniques to compare the repair of both intra- and interstrand crosslinks in specific actively transcribing genes [dihydrofolate reductase (DHRF), multidrug resistance (*mdr*1) and c-*myc*] *vs* non-coding fragments of DNA. Intrastrand adducts were detected by bacterial (A)BC exinuclease enzyme digestion and interstrand crosslinks via a denaturation-renaturation procedure followed by gel electrophoresis and Southern hybridisation. He showed, using two cisplatin sensitive and acquired-resistant pairs of human ovarian cancer cell lines (A2780/DDP and 2008/DDP) that removal of intrastrand adducts was similar in both the parent and resistant lines. Removal of interstrand crosslinks, however, was much faster from specific genes in the resistant lines as compared to the sensitive counterparts. Interestingly, alkaline elution revealed no differences in removal. Thus cisplatin resistance can be associated with an increase in gene-specific DNA repair without marked changes in the overall genome repair. Instead of using (A)BC exinuclease, Eastman (Dartmouth School of Medicine, Hanover, New Hampshire, USA) described a polymerase chain reaction-based method of similar sensitivity to detect cisplatin adducts in DNA.

Using clinical samples from patients receiving platinum-based chemotherapy, Reed (National Cancer Institute, Bethesda, Maryland, USA) assessed RNA for the human DNA excision repair gene ERCC1. High levels of expression in fresh tumour tissue appeared directly related to clinical resistance ($P = 0.051$). In one patient where levels in tumour tissue and peripheral blood were measured before treatment with carboplatin and after the development of clinical resistance, ERCC1 expression increased 10-fold in the tumour and 5-fold in white blood cells. The group suggested that ERCC1 expression may be an important marker for clinically relevant DNA repair capability. Wood (Imperial Cancer Research Fund, Potters Bar, Hertfordshire, UK) described an assay which measures the repair of cisplatin-damaged DNA *in vitro* by human cell extracts. Preliminary attempts to apply the assay to investigate DNA repair capacity in human tumour biopsies were described by Harris (Churchill Hospital, Oxford, UK). Further evidence for the involvement of DNA repair capacity in cisplatin resistance was described by Harrap (Institute of Cancer Research, Sutton, Surrey, UK) using a cisplatin-hypersensitive human testicular nonseminomatous germ cell line and a 6.5-fold acquired resistant variant, the removal of total platinum bound to DNA being enhanced in the resistant line.

The role of oncogene expression and signal transduction pathway regulation on cisplatin sensitivity was addressed by several groups. Sklar (University of Michigan, Ann Arbor, Michigan, USA) described the modulation of resistance to cisplatin in mouse NIH3T3 fibroblasts by c-Ha-*ras* and c-*myc* oncogenes. *Ras* oncogene transfection significantly increased resistance to cisplatin (4.5-fold to 10-fold differences in $IC_{50}$ values) as well as to ionising radiation. Using cells containing amplified c-*myc* sequences in either sense or antisense orientations, the group showed that cisplatin resistance correlated with c-*myc* levels; induction of c-*myc* increased cisplatin resistance by about 3-fold. Howell (UCSD, La Jolla, California, USA) showed that activiation of either the protein kinase A or protein kinase C pathways enhanced sensitivity of human ovarian 2008 cells to cisplatin by 2- to 3-fold. In contrast, and in accordance with the Sklar studies, c-Ha-*ras* oncogene over-expression in NIH3T3 cells (greater than 10-fold increase in $p21^{ras}$ expression after 24 h) reduced cisplatin sensitivity by 8.2-fold. Scanlon (City of Hope National Medical Center, Duarte, California, USA) described studies suggesting a role for the c-*fos* oncogene in cisplatin resistance using a *fos* ribozyme (catalytic RNA) expressed in the A2780 human ovarian carcinoma cell line. However, not all studies showed such effects. Kerr (University of Glasgow, Glasgow, UK) transfected H-*ras* and c-*myc* into mink lung epithelial cells and showed no alterations in sensitivity to cisplatin.

Essigmann (Massachusetts Institute of Technology, Cambridge, Massachusetts, USA) described further studies on the recently discovered protein present in human and rodent cells that specifically binds to DNA modified with cisplatin but does not bind to inactive platinum agents (e.g. transplatin). The protein, of an apparent molecular weight of 91,000, maps to chromosome 11q and binds specifically to the 1,2-intrastrand G-G and A-G adducts. Although expression has been found in many different tissues it did not correlate with cisplatin resistance in a number of human, hamster and mouse cell lines. Other such damage recognition proteins, which appear from preliminary experiments to be overexpressed in cisplatin resistant cell lines, were described by Brown (University of Glasgow, Glasgow, UK) and Chao (Chang Gung Medical College, Taoyman, Taiwan).

There was one report of the isolation of a gene associated with cisplatin resistance (Enns, UCSD, La Jolla, California, USA). Libraries were made from a human ovarian cell line (2008) and a 10-fold cisplatin resistant variant (C13) and cotransfected with PSP2-neo into Chinese hamster ovary cells. Human inserts from seven cisplatin-resistant clones were recovered using polymerase chain reaction methodology. All of the inserts were of the same size; two were sequenced revealing a sequence corresponding closely to the human mitochondrial heat shock protein 60, P1. To date, however, the gene has not been transfected back into the parent line and consequently its biological relevance to cisplatin resistance is uncertain.

### Clinical trials

Early phase clinical trials of eight new antitumour platinum complexes were reported. Their chemical structures are shown in Figure 4.

Christian (National Cancer Institute, Bethesda, Maryland, USA) and O'Rourke (Brooke Army Medical Center, Houston, Texas, USA) presented preliminary reports of Phase I studies of ormaplatin (tetrachloro(l-trans)1,2-diaminocyclohexane platinum (IV)). This DACH compound with pharmaceutical properties suitable for intravenous formulation lacks cross resistance against acquired cisplatin resistant murine leukaemias and has a preclinical antitumour spectrum otherwise similar to cisplatin. Maximum tolerated doses and dose limiting toxicities have not yet been reached, but emesis has been encountered at the lowest dose levels.

The development of oxaliplatin (1,2-diaminocyclohexane(l-trans)oxalatoplatinum (II)) was reviewed by Misset (Hospital Paul-Brousse, Villejuif, France). This agent has activity against cisplatin resistant L1210 and less experimental nephrotoxicity and myelosuppression than cisplatin. Phase I studies of intravenous bolus dose and continuous intravenous infusion oxaliplatin found emesis and cumulative neurotoxicity to be dose limiting. The neurotoxicity was characterised by progressive sensory neuropathy, acute parasthesiae and electrophysiological abnormalities. Phase II studies are ongoing in France.

Perez-Solar (MD Anderson Cancer Center, Houston, Texas, USA) reviewed the development of cis-bis-neodecanoato-trans-R,R-1,2-diaminocyclohexane platinum (II) (NDDP), a platinum complex with high liposomal entrapment efficiency and stability. In liposomal formulation (L-NDDP) this agent has partial non-cross resistance in cisplatin resistant L1210, superior activity to cisplatin in experimental hepatic metastases, and less nephrotoxicity than cisplatin in mice. A phase I study of intravenous L-NDDP found a maximum tolerated dose of 312.5 mg $m^{-2}$ and dose limiting myelosuppresion. No nephrotoxicity or neurotoxicity was encountered, but emesis occurred in most patients despite prophylactic antiemetics. A Phase I study of L-NDDP administered by hepatic arterial infusion is ongoing.

**Figure 4** Platinum complexes currently in Phase I/II development.

Zeniplatin (2,-bis(aminomethyl)-1,3-propanediol-N,N' (1,1-cyclobutanedicarboxylato(2-)-0,0'platinum(I)) and enloplatin ((sp-4-2)-(1,1-cyclobutanedicarboxylato(2-)-0,0')tetrahydro-4H -pyran-4,4methamine N,N) platinum(II) are being developed by the American Cyanamid Company. Both are highly water soluble complexes with experimental *in vivo* antitumour activity broadly comparable to cisplatin and carboplatin, with the exception of enloplatin's non-cross resistance against cisplatin resistant L1210. Both compounds appear less nephrotoxic than cisplatin on the basis of BUN elevations in rats. Ceulemans (Univeristy Catholique de Louvain, Brussels, Belgium) reported a Phase I study of enloplatin in which neutropenia and nephrotoxicity were dose limiting at the maximum tolerated dose of 1227 mg $m^{-2}$. De Valeriola (Institut Jules Bordet, Brussels, Belgium and University of Maryland Cancer Center, Baltimore, Maryland, USA) reported a Phase I study of zeniplatin and the maximum tolerated dose was 145 mg $m^{-2}$, with dose limiting leukopenia. Phase II studies of zeniplatin were presented by Jones (Royal Marsden Hospital, Sutton, Surrey, UK) and Aamdal (EORTC Early Clinical Trials Group). This agent is active in non-small lung cancer and malignant melanoma, though nephrotoxicity appears to be a major limitation.

Majima (Institute of Microbiol Chemistry, Tokyo, Japan) reviewed the Japanese development of three platinum complexes, 254-S (cisdiammine(glycolato)platinum II), Cl 973 or NK 121 ((SR-4-3-1R)-[1,1-cyclobutanedicarboxylato(2-)] (2 methyl-1,4-butanediamine-N,N')platinum II), and DWA2114R (2 - aminomethyl - pyrrolidine (1,1 -cyclobutanedicarboxylato) platinum II). All three have antitumour activity and low nephrotoxic potential in murine models, activity against human tumour xenografts, and partial or complete non-cross resistance against cisplatin resistant murine K562 leukaemia. In Phase I studies, including that presented by O'Dwyer (Fox Chase Cancer Center, Philadelphia, Pennsylvania, USA), leukopenia or thrombocytopenia were the dose limiting toxicities in all instances, except in the case of continuous intravenous infusion of DWA2114R, when emesis and diarrhoea were dose limiting. Phase II studies are ongoing in Japan. Hirabayashi (National Hospital Fukuyama, Fukuyama, Japan) reported encouraging activity of 254-S in combination with peplomycin and ifosfamide in advanced cervical cancer.

After the clinical evaluation of these eight new platinum complexes have been completed little doubt should exist as to the utility of the cisplatin resistant murine leukaemias and the 1,2-diaminocyclohexane ligand, as models of and a strategy to circumvent clinical cisplatin resistance, since seven of these new complexes lack cross resistance to cisplatin resistant murine leukaemias, and three are 1,2-diaminocyclohexane derivatives. The dicarboxylatocyclobutane substituent is a feature of four platinum complexes in clinical development. Two of these agents are associated with significant clinical nephrotoxicity which is surprising in view of the previously described relationship between leaving group stability and kidney damage. Some of the new compounds cause major non-haematological toxicities, e.g. neurotoxicity, nephrotoxicity and severe emesis, which may limit their clinical application unless unique activity is demonstrated. Interestingly, some of the new platinum complexes are unlike carboplatin in that their myelosuppression predominants against the white cell lineage rather than platelets.

Taxol, an alkaloid extracted from the Pacific Yew (*Taxus brevifolia*) is active in advanced and cisplatin refractory ovarian cancer, and has major toxicities that are not shared with cisplatin. The cytotoxicity of the taxol-cisplatin combination is sequence dependent, with taxol followed by cisplatin treatment sequence having the highest *in vitro* activity. A phase I study of this combination was reported by Rowinsky (Johns

Hopkins Oncology Center, Baltimore, Maryland, USA). Neutropoenia was dose limiting and sequence dependent, being more severe with cisplatin preceding taxol. This treatment sequence was also associated with reduced taxol clearance. Other toxicities include emesis, alopecia and, in four patients, ventricular tachycardia. The recommendations for Phase II studies are the taxol before cisplatin treatment sequence and cisplatin/taxol doses of 75–135 mg $m^{-2}$ with further taxol escalation if tolerable. Results of these studies are awaited with interest.

The development of ORG 2766, a neurotrophic ACTH analogue devoid of adrenocorticotrophic properties was updated by Hamers (Rudolf Magnus Institute, Utrecht, The Netherlands). This peptide's efficacy in the prophylaxis and treatment of cisplatin-induced neuropathy was first demonstrated in the rat model and its preventive action has been confirmed in ovarian cancer patients receiving cisplatin, in whom very few side effects were apparent. Experiments demonstrating the efficacy of ORG 2766 during high dose cisplatin treatment were presented.

**Reference**

*Anti-Cancer Drug Design* (1991) **6**, 211.